# OES diagnostics as a universal technique to control the Si etching structures profile in ICP

**DOI:** 10.1038/s41598-022-09266-x

**Published:** 2022-03-28

**Authors:** Artem A. Osipov, Gleb A. Iankevich, Anastasia B. Speshilova, Alina E. Gagaeva, Armenak A. Osipov, Yakov B. Enns, Alexey N. Kazakin, Ekaterina V. Endiiarova, Ilya A. Belyanov, Viktor I. Ivanov, Sergey E. Alexandrov

**Affiliations:** 1 Peter the Great St. Petersburg Polytechnic University, St. Petersburg, Russian Federation 195251; 2 Institute of Nanotechnology, Karlsruhe Institute of Technology, Hermann‑von‑Helmholtz‑Platz 1, 76344 Eggenstein‑Leopoldshafen, Germany; 3 Institute of Mineralogy of Southern-Urals Federal Research Center of Mineralogy and Geoecology of Ural Branch of RAS, Miass, Chelyabinsk Region Russian Federation 456317

**Keywords:** Materials for devices, Techniques and instrumentation

## Abstract

In this work, we demonstrate the high efficiency of optical emission spectroscopy to estimate the etching profile of silicon structures in SF_6_/C_4_F_8_/O_2_ plasma. The etching profile is evaluated as a ratio of the emission intensity of the oxygen line (778.1 nm) to the fluorine lines (685.8 nm and 703.9 nm). It was found that for the creation of directional structures with line sizes from 13 to 100 μm and aspect ratio from ≈ 0.15 to ≈ 5 the optimal intensities ratio is in the range of 2–6, and for structures from 400 to 4000 μm with aspect ratio from ≈ 0.03 to ≈ 0.37 it is in the range 1.5–2. Moreover, the influence of the process parameters on the etching rate of silicon, the etching rate of aluminum, the inclination angle of the profile wall of the etched window, the selectivity of silicon etching with respect to aluminum, and the influence on the overetching (Bowing effect) of the structure was investigated.

## Introduction

For decades, there has been tremendous interest in the development and creation of microelectromechanical systems (MEMS), devices that combine microelectronic and micromechanical components^1^. The development of MEMS is essential for the manufacture of highly sensitive inertial sensors, micro-capacitors, accelerometers, gyroscopes, micro-optical elements, etc^2–6^. The key step in the production and development of modern MEMS is the precision of the pattern transfer to a silicon substrate, with the creation of vertical structures with a high aspect ratio^7–10^. The main approach to achieve this goal is to have the surface reaction occur only in the vertical direction^11^.The importance of obtaining structures with a high aspect ratio is due to the need to integrate an increasing number of individual microdevices into one complex microsystem to perform a variety of functions. In this regard, a new requirement began to be imposed on microdevices—not only accuracy in two dimensions, but also expansion into the third dimension^12^.

There are two main technologies for deep anisotropic silicon etching: the Bosch process and cryogenic etching. Both technologies were developed to provide high-rate vertical etching of profiles with a high aspect ratio^9^. The most popular method is the Bosch process, which is based on the alternation of the etching and sidewall passivation steps to achieve directional etching^13–15^. During the passivation step, the dissociation products of the passivation gas in plasma (e.g., C_4_F_8_) are deposited on the substrate, resulting in the formation of a conformal protective layer on the exposed silicon areas. At the etching step, the etching gas (e.g., SF_6_) is also decomposed by plasma into ions, radicals, and chemically active particles (CAP) to etch silicon. After removal of the polymer layer from the bottom of the structure due to the ions, the radicals cause spontaneous and isotropic etching of silicon by adsorption followed by the formation and desorption of volatile products such as SiF_4_. Thus, the complete deposition/etching cycle can be divided into three distinctive but sequential steps: polymer deposition, polymer removal from the bottom surface and isotropic silicon etching. The main advantages of the Bosch process are the high etch rate, high etch selectivity with respect to the mask material, good anisotropy control, and the fact that the temperature of the substrate holder is in the range from 0 to 15 °C during the etching^12^. However, this process has certain drawbacks such as the residual contamination of the system reactor walls by the polymer formed during the passivation stage and the serrated profile of the sidewalls of the etched structure. The last drawback can negatively affect the results of the subsequent filling of the etched structure with other materials^16^.

An alternative to the Bosch process is a continuous cryogenic process in which etching is usually performed at a low temperature (from − 130 to − 100 °C) using an SF_6_/O_2_ mixture^17^. The F* radicals formed in the plasma discharge are used for the isotropic etching of silicon. The oxygen radicals serve for surface oxidation of silicon, leading to the growth of a passivating SiO_x_F_y_ layer, which protects the sidewalls of the structure from etching. When the wafer is brought to room temperature, this passivation layer desorbs naturally. And due to the absence of etching/passivation modes switching, the continuous Cryo process provides vertical sidewalls without noticeable roughness. However, the etching rate of the cryogenic process is several times slower compared to the Bosch process, so there is a possibility of photoresist cracking under the influence of low temperatures during a long process^18^.

It is worth noting that both technologies require complex equipment. The Bosch process is characterized by rapid gas switching and requires an expensive generator to match this power load, while the cryogenic process is conducted at ultra-low temperatures. Recently, the CORE process was developed for silicon etching. This process includes 4 steps: (1) purging the reactor and etching of the surface from unwanted contaminants and plasma particles that may have formed on the surface during the etching step in the fluorine plasma, (2) forming an oxide film on the exposed silicon surface to provide directionality to the etching process, (3) removing the oxide film from the bottom of the structure, and finally (4) actual etching of the silicon, after which the cycle is repeated. The advantage of this technique is the replacement of C_x_F_y_ gases with oxygen. Oxygen passivation, unlike C_x_F_y_, is a self-limiting process and, therefore, the oxide thickness at the bottom of the etching window will be approximately the same for structures with different aspect ratios^19–21^. The key to the realization of such a process is to carry it out in a system that uses flat electrodes for plasma generation (capacitive coupling). Otherwise, there is a high risk of AlF_x_ formation (with inductive coupling, without Faraday screen) during the etching process. The disadvantage of the process is the low etching rate of silicon.

Of particular interest are the one-step processes for creating deep directional structures in silicon at classical facilities at room temperature with inductively coupled plasma (ICP) sources. The advantage of such processes is the simplicity of the equipment (compared with equipment that used in the above-mentioned processes), an acceptable etching rate and the absence of alternating steps, which has a positive effect on the roughness of the walls of the obtained structures. Many works have been devoted to the mixed process, both in SF_6_/C_x_F_y_ plasmas and in plasmas where oxygen is used for passivation^22–28^. Clearly, when using an oxygen-added gas mixture for silicon etching, it is important to choose the optimal O/F ratio, which, for the reasons mentioned above, will affect both the etching rate, the geometric parameters, and roughness of the etched structures. Monitoring of the O/F ratio can be carried out, in particular, by optical emission spectroscopy (OES). However, as far as we know, in the literature there are no studies devoted to the diagnostics of plasma in single-stage processes by the OES method, aiming to determine the optimal O/F ratio that would provide a vertical etch profile. In this regard, this work is focused on the study of mixed etching process using OES, in order to determine the correlation between the O/F ratio value and the parameters of silicon etching process in SF_6_/C_4_F_8_/O_2_ gas mixture plasma.

## Experimental details

**Figure 1:**
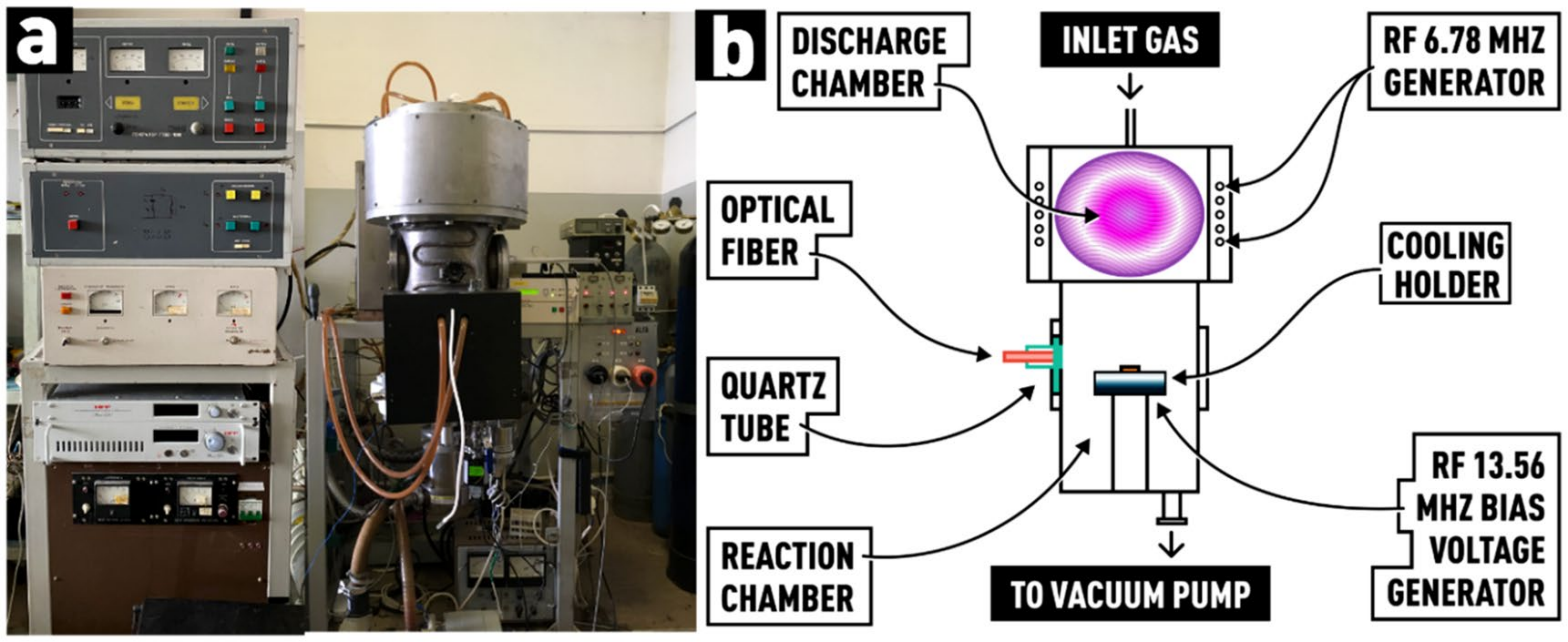
(**a**) General view of the PCE system; (**b**) schematic drawing of the processing and plasma chambers.

The experiments were performed on a custom-built plasma-chemical etching (PCE) system with an ICP source (Fig. 1a). As can be seen from Fig. 1b, the reactor of the system consisted of two main chambers—discharge and reaction. Plasma in the discharge chamber (diameter 23 cm, height 22.5 cm) is generated by applying high-frequency (HF) power to the inductor coil of specific geometry from a HF generator (f = 6.78 MHz, W_max_ = 4000 W) through a resonance matching device. The plasma generated in the discharge chamber diffusively propagates to the reaction chamber. The reaction chamber (diameter 23 cm, height 33.5 cm) of cylindrical shape was made of stainless steel AISI 321. To induce a bias potential on the substrate holder, an RF voltage of 13.56 MHz from a separate RF generator was applied to it.

The plasma generator inductor is located outside the process chamber and is isolated from it by a wall made of alumina ceramics, on which zirconium oxide was deposited. To minimize the erosion of the reactor walls and reduce the plasma potential, a multi-slit electrostatic shield is installed between the inductor and the ceramic reactor wall. A gas shower is integrated into the upper water-cooled flange of the reactor. A substrate holder, cooled by continuous cold-water flow for efficient heat removal, is located in the lower part of the plasma generator.

Optical emission spectra were recorded using an OceanOptics HR 4000 spectrometer in the wavelength range of 200 to 1120 nm with a resolution of ~ 0.02 nm. The spectrometer was paired with the system using a fiber-optic cable for transmitting the plasma radiation to the spectrometer's entrance slit. The fiber-optic cable is located in close proximity to the viewport window (made of quartz) located on the side-flange of the system (Fig. 1b). The spectra were processed using SpectraGryph 1.2.14 software.

**Figure 2:**
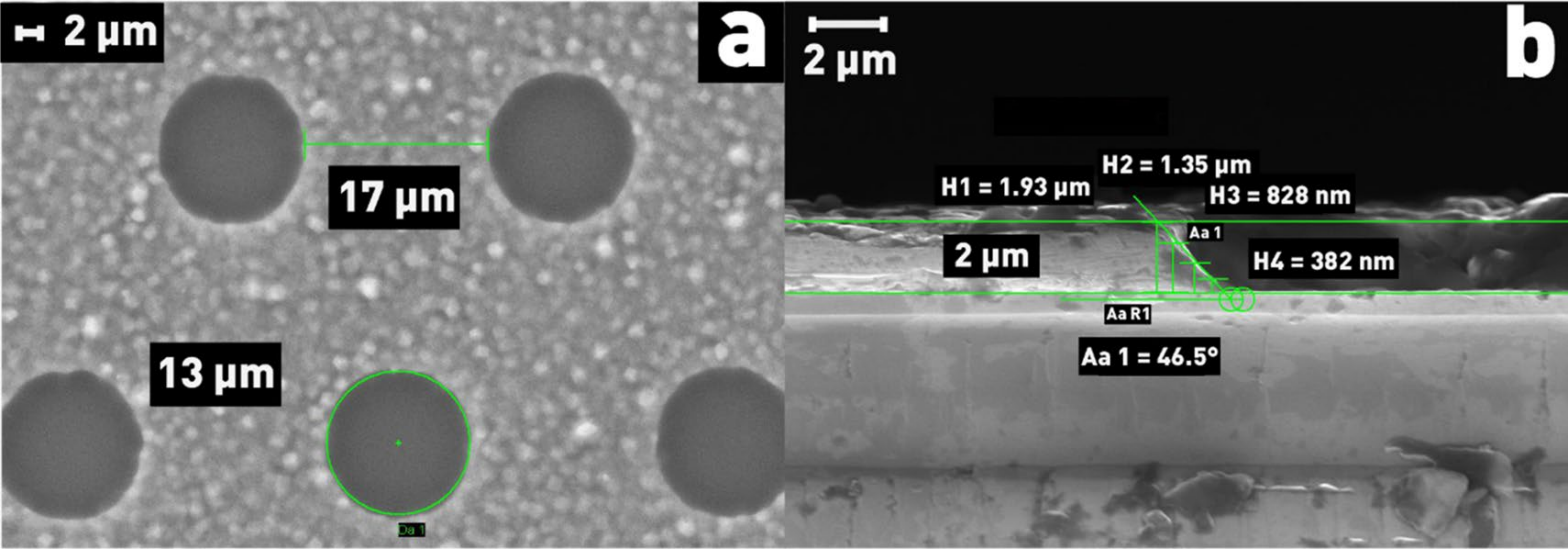
(**a**) Deposited mask (top view); (**b**) sideview of the windows in deposited Al mask.

Single-side polished monocrystalline silicon (n-type, P-doped) wafers of 76 mm in diameter and 380 µm in thickness were used as etching samples. A 2 μm-thick aluminum mask was deposited on the wafers (Fig. 2).

In all experiments, samples were cleaned sequentially with acetone, ethyl alcohol, and deionized water for 10 min before starting the PCE process. In addition, after loading the Si sample into the reaction chamber prior to the experiment, the samples were treated in argon plasma for 10 min in order to remove residual contaminants from the substrate surface. The parameters of the cleaning process in argon were as follows: The RF power (W) applied to the discharge chamber inductor was 1000 W, the bias voltage (U_bias_) on the substrate holder was -35 V, the pressure in the reaction chamber (P) was 0.75 Pa, and the argon flow rate (Q_Ar_) was 21.75 sccm.

After each etching process, the sample was unloaded, and the chamber was cleaned by oxygen plasma for 30 min. This operation was performed in order to remove the undesirable contaminants and volatile reaction products generated by the previous PCE process from the chamber surface. These contaminants can redeposit on the chamber walls and introduce deviations during the subsequent OES registration of the Si etching processes.

Sulfur hexafluoride (SF_6_) of high purity 99.998% (GOST TU 6-02-1249-83) was used as the main etchant gas. Oxygen (O_2_ class 6.0, 99.9999%, GOST TU 2114-001-05798345-2007) and octafluorocyclobutane C_4_F_8_ (GOST TU 2412-128-05807960-96) were used as additive gases.

The etch profile depth, residual mask thickness, structure overetching (Bowing effect), and the wall angle of the etch window profile were determined from microphotographs obtained using a CarlZeiss Supra 55VP scanning electron microscope with an accuracy of ± 2.5%. Energy-dispersive X-ray spectroscopy (EDX) was used to determine the composition of the protective film formed during etching on silicon.

## Results and discussion

**Figure 3:**
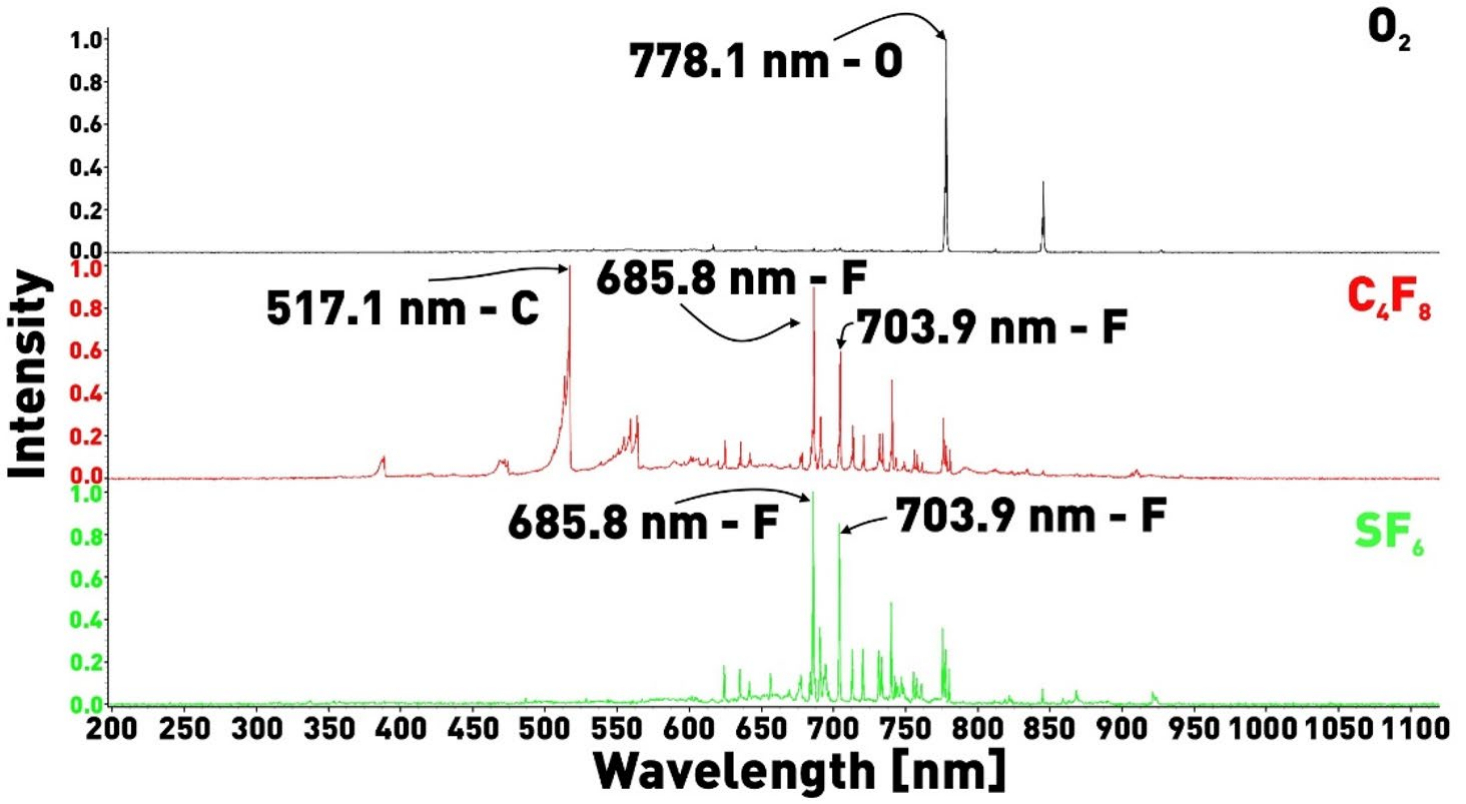
ICP OES of pure O_2_, SF_6_, C_4_F_8_.

Figure 3 shows the emission spectra of pure O_2_, C_4_F_8_, and SF_6_ plasmas. In the OES spectrum of oxygen plasma, the most intense line is observed near 778.1 nm (2s^2^2p^3^(^4^S°)3s^5^ $S_{2}^{ \circ }$ → 2s^2^2p^3^(^4^S°)3p^5^p_1_ transition). In the OES spectra of C_4_F_8_ and SF_6_ plasmas, the most intense fluorine lines, 685.8 and 703.9 nm, are associated with s^2^2p^4^(^3^P)3p:^4^D°_7/2_ → 2s^2^2p^4^(^3^P)3s:^4^P_3/2_ and 2s^2^2p^4^(^3^P)3p:^2^P°_3/2_ → 2s^2^2p^4^(^3^P)3s:^2^P_3/2_ transitions of excited fluorine atoms, respectively^29^. Assuming that the intensities of these lines are proportional to the concentrations of oxygen and fluorine atoms in the gas discharge, we can introduce a parameter:

1 $$Z = \frac{{I_{O2} }}{{I_{F1} + I_{F2} }}$$

characterizing the O/F ratio. In this formula, I_O2_ is the oxygen emission intensity at 778.1 nm, I_F1_ and I_F2_ are the fluorine emission intensities at 685.8 nm and 703.9 nm, respectively.

**Figure 4:**
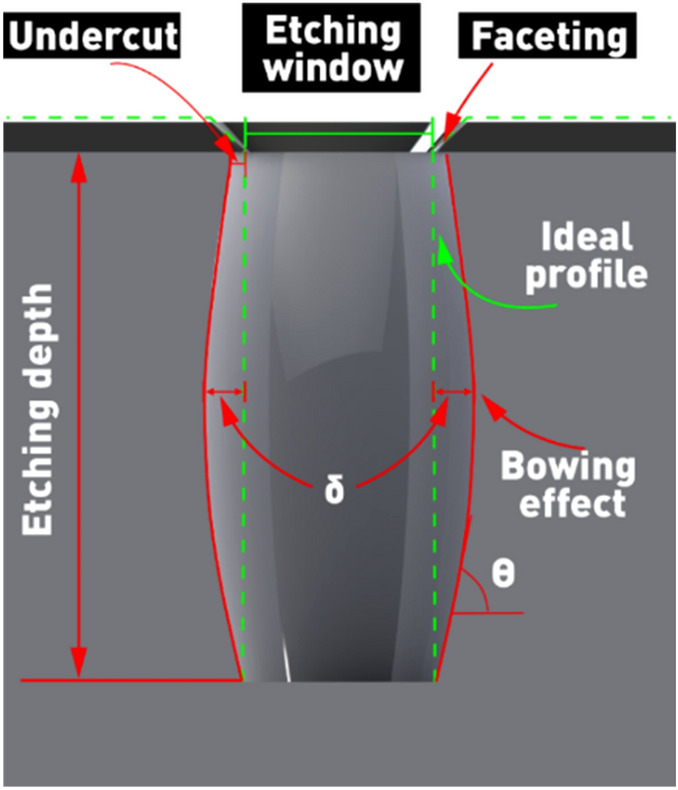
Schematic of measurements of the inclination angle and “bowing effect” of the etched structure.

Before describing the results and discussing them, it is important to focus on Fig. 4, which explains some of the specific defects that occur during the Si PCE process.

In the PCE process, the directionality of the etching is provided by the ions generated in the plasma and accelerated in the direction perpendicular to the substrate surface plane^16^. The directional movement of the ions provides higher vertical etching rates compared to the etching rates of the side surfaces of the treated windows. However, during the etching process, not only the surface of the desired material (Si) to be removed is subjected to ion bombardment, but also the masking material—for example, aluminum. As a result, the aluminum mask is subject to erosion accompanied with the faceting effect^16^ shown in Fig. 4. The nature of this effect is the higher etching rate of the masks’ edge compared to its surface. This is due to the fact that the edge of the mask is in contact with a larger spatial plasma volume and, it is subjected to a larger flux of active particles compared to the horizontal or vertical (sidewalls) surfaces^28,30,31^. As a result, the etched window grows in cross-sectional dimensions. In this work, we measured the total "Bowing effect" of the structure, not the "undercut" under the mask. The problem of mask etching is also illustrated in Fig. 4. The etching of silicon occurs not only deeper into the wafer, but also to the sides of the window—under the mask, due to chemical reactions with active plasma particles.

Another negative event observed in some of the experiments is the “bowing effect”—the formation of a barrel-shaped etched profile, which can be numerically characterized by the value of the maximum deviation of the sidewall contour from the vertical, (δ). Such profile shape is caused by a change in the angular distribution of the ions as they move deeper into the etched structure^32,33^. In the upper part of the etching window, ions move at different angles (directions) and, depending on the energy of the ions, a fairly large "undercut" of the structure can be observed. However, as the etching depth increases, the number of ions etching the sidewall is drained, and due to the shadowing effect (blocking of incoming ions by the opposite sidewall) at a certain critical angle the ions stop etching the sidewall. In other words, in the lower part of the etching window, the sidewall etching by ions stops, and only the bottom surface of the profile is etched by ions moving at an angle close to the normal to the surface of the substrate. As a result, a barrel-shaped etch profile is formed. To determine the wall angle of the etched window profile in this case, we used the angle, Θ, between the contour of the sidewall and the substrate plane (Fig. 4).

### Influence of gases mixture composition

The etching rate primarily depends on the concentration of chemically active plasma particles (CAP), so one of the important parameters affecting the rate of the Si PCE process is the process gases flow. Variation of the CAP composition can be used as a way to control selectivity. In addition, one way to achieve anisotropic etching is to reduce the flux of fluorine atoms in the plasma, as well as to involve the passivation layer deposited on the silicon surface and protecting the sidewalls of the structure from the reactive particles^12,34,35^.

#### Sulfur hexafluoride (SF_6_)

**Figure 5:**
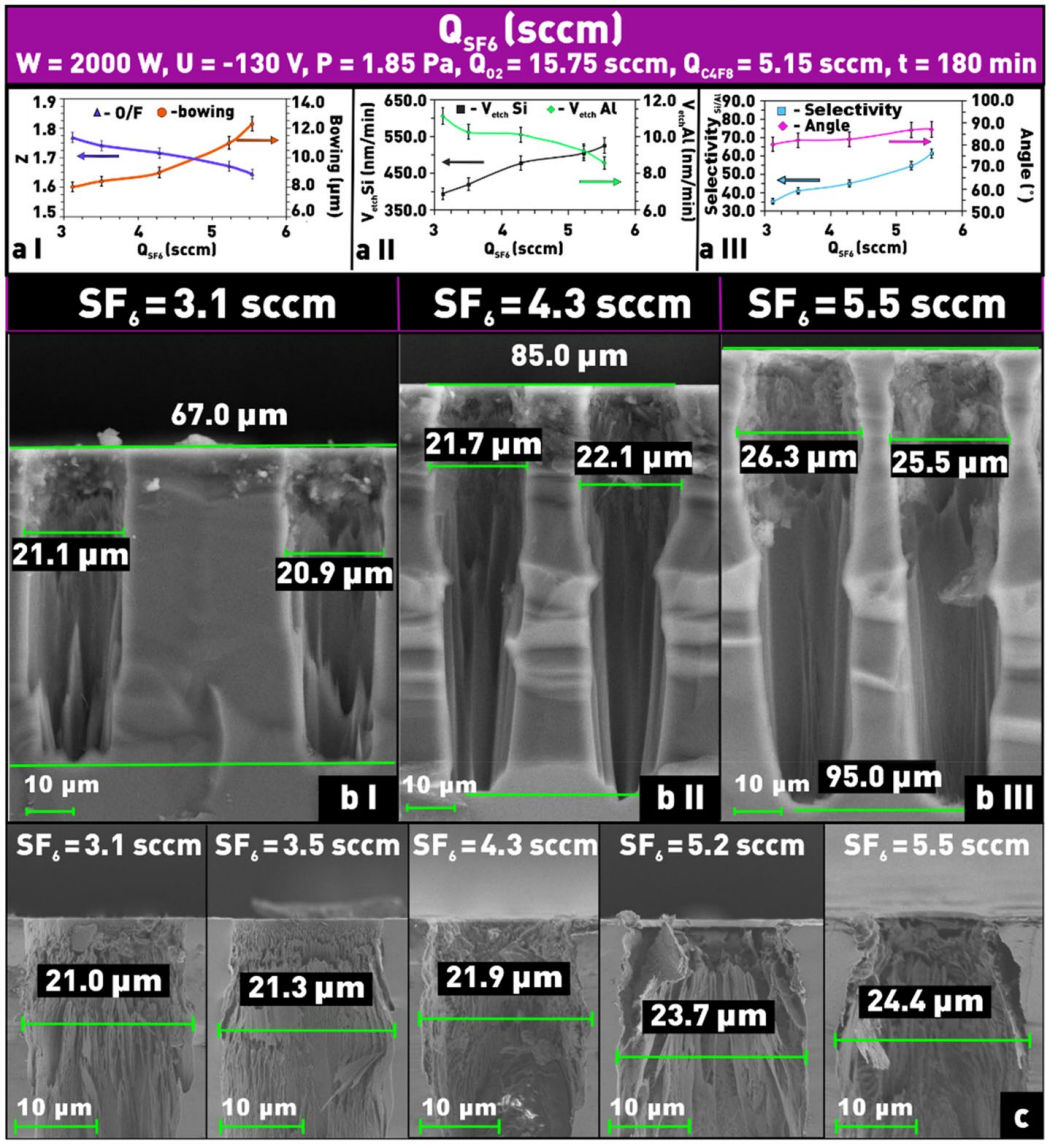
Dependences on SF_6_ flow rate in SF_6_/C_4_F_8_/O_2_ plasma: (**a I**) of δ and Z; (**a II**) of etching rates of silicon and aluminum; (**a III**) of etch selectivity and angle Θ; (**b I–III**) microphotographs of etched windows profiles at different contents of SF_6_ in SF_6_/C_4_F_8_/O_2_ plasma; (**c**) microphotographs of etched windows at different contents of SF_6_ in SF_6_/C_4_F_8_/O_2_ plasma to evaluate the structure overetching (δ).

To determine the effect of SF_6_ content in the total gas mixture on the result of the Si PCE process, a series of experiments at different SF_6_ flow rates and fixed values of other process parameters were performed. The obtained results (Fig. 5a II,b I–III) show that the increase in SF_6_ flow rate in the total gas mixture leads to an increase in the etching rate. Obviously, the observed increase in the etching rate is associated with an increase in the concentration of active fluorine particles in the process chamber^36^. In its turn, a higher concentration of fluorine radicals leads to an increase in the contribution of the chemical part of the PCE process over the physical part (sputtering), which is strongly evidenced by the increase in selectivity (Fig. 5a III). As can be seen from Fig. 5a II the increase in the etching selectivity is due to both an increase in the Si etch rate and a decrease in the Al mask etch rate. Moreover, the increase of fluorine radical concentration is evidenced by the almost 1.5 times increase of the “bowing effect” (from 8 to 12.3 μm), when the SF_6_ flow increased from 3.1 sccm to 5.5 sccm (Fig. 5a I,b I–III,c), as well as the decrease of the Z parameter. So, the combination of the above-mentioned facts clearly indicates an increase in the chemical part role in the Si PCE process with an increase in the SF_6_ flow rate.

#### Octafluorocyclobutane (C_4_F_8_)

**Figure 6:**
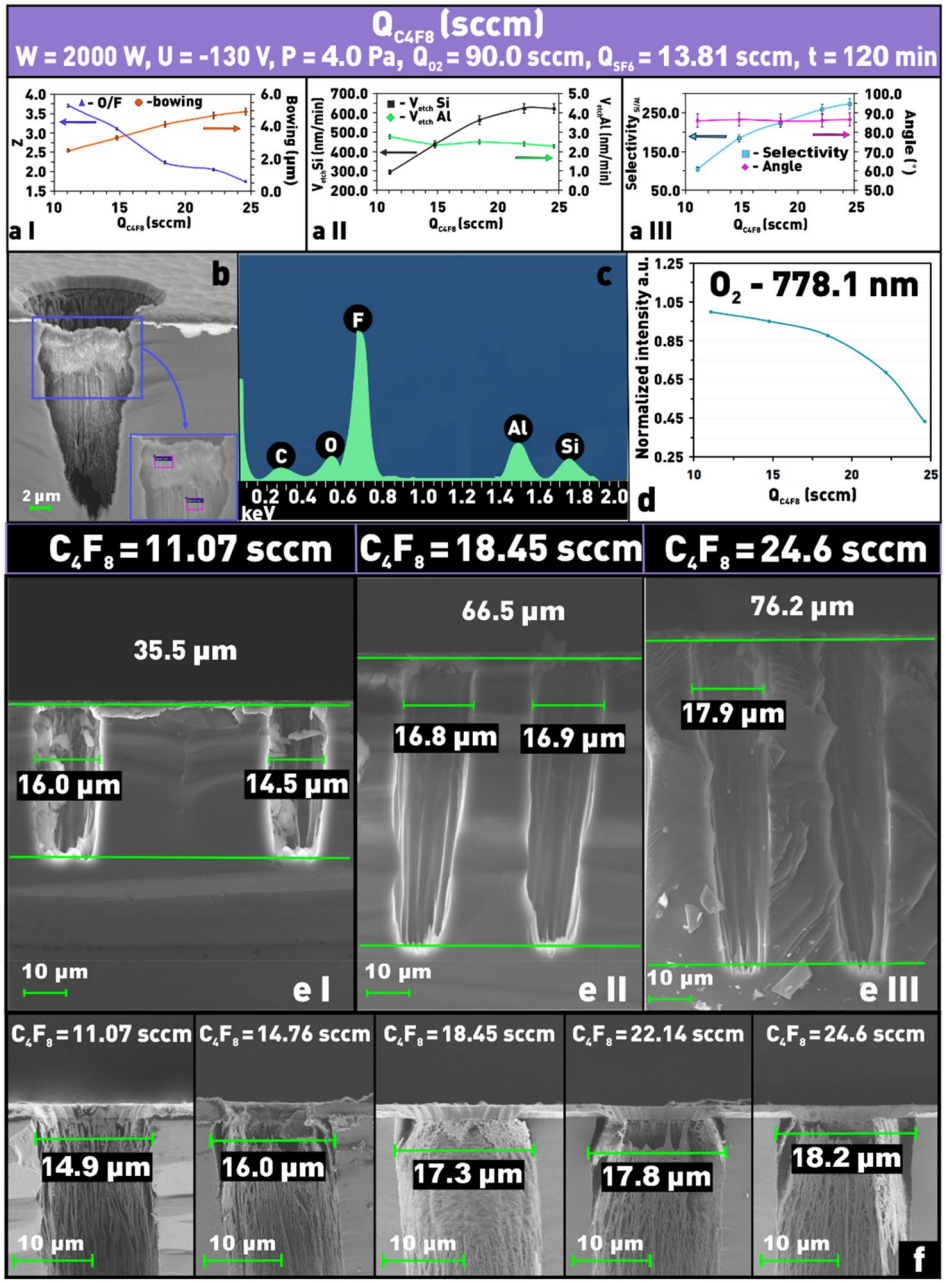
Dependences on C_4_F_8_ flow rate in SF_6_/C_4_F_8_/O_2_ plasma: (**a I**) of δ and Z; (**a II**) of silicon and aluminum etching; (**a III**) of etch selectivity and Θ angle; (**b**) microphotograph of protective layer after the PCE process; (**c**) EDS of protective film; (**d**) intensity of 778.1 nm line as a function of C_4_F_8_ flow rate; (**e I**–**III**) microphotographs of etching profiles at different contents of C_4_F_8_ in SF_6_/C_4_F_8_/O_2_ plasma; (**f**) microphotographs of etched windows at different contents of C_4_F_8_ in SF_6_/C_4_F_8_/O_2_ plasma to evaluate the structure overetching (δ).

The increase of C_4_F_8_ consumption in the total SF_6_/C_4_F_8_/O_2_ gas mixture has a similar effect on the Si etching rate as in the case of SF_6_. So, with the increase of the flow rate of C_4_F_8_ more than twice, the etching rate of silicon increases from ≈ 300 to ≈ 630 nm/min (Fig. 6a II,e I–III). It is known that in SF_6_/C_4_F_8_ gas mixture-based plasma without oxygen addition, an increase in C_4_F_8_ content usually leads to a decrease in the structure “bowing effect” and even to a narrowing of the etching window bottom due to the formation of a protective polymer layer C_x_F_y_^23,37,38^. In the SF_6_/C_4_F_8_/O_2_ based plasma the opposite effect is observed. This is probably due to the fact that the amount of free oxygen radicals, which also participate in the passivation of Si etch window walls, sharply decreases with increasing C_4_F_8_ consumption. And at the same time, the concentration of F* radicals increases. According to the OES data (Fig. 6d), the intensity of the 778.1 nm oxygen emission line decreases more than twice as the C_4_F_8_ flow rate increases from 11.07 to 24.6 sccm, indicating a decrease in the concentration of free oxygen radicals in the plasma. In addition, qualitative EDX analysis of the composition of the protective film (Fig. 6b,c) formed during Si PCE in the SF_6_/C_4_F_8_/O_2_ gas mixture showed the presence of Si, F, O, C, and Al in it. The presence of Al is obviously associated with the sputtering of the protective mask and its redeposition on the treated window surface. The presence of such elements as Si, F, and O allows us to assume that the formed passivating film is a SiO_x_F_y_ type of compound^26^. Finally, the presence of carbon in the protective film allows the formation of the C_x_F_y_ polymer during the etching.

Further, it should be noted that as the C_4_F_8_ flow rate increases, the parameter δ characterizing the deviation of the etching contour from the ideal vertical increases twice (Fig. 6a I,f), from 2.5 to 5 µm. As well as the selectivity of Si with respect to the mask material which also increases by more than 2.5 times. As follows from Fig. 6a III, the increase in selectivity is associated with an increase in the Si etch rate, since the Al etch rate remains nearly constant over the entire range Q_C4F8_ variation.

All of the above occurs on the background of the Z parameter decrease. In general, the determined patterns are qualitatively similar to those observed when varying the value of Q_SF6_ and described in the previous section. It follows that increasing the C_4_F_8_ flow rate in the SF_6_/C_4_F_8_/O_2_ mixture has a similar effect on the etching process parameters as Q_SF6_, namely, it leads to an increase in the chemical part of the PCE process.

#### *Oxygen (O*_*2*_*)*

**Figure 7:**
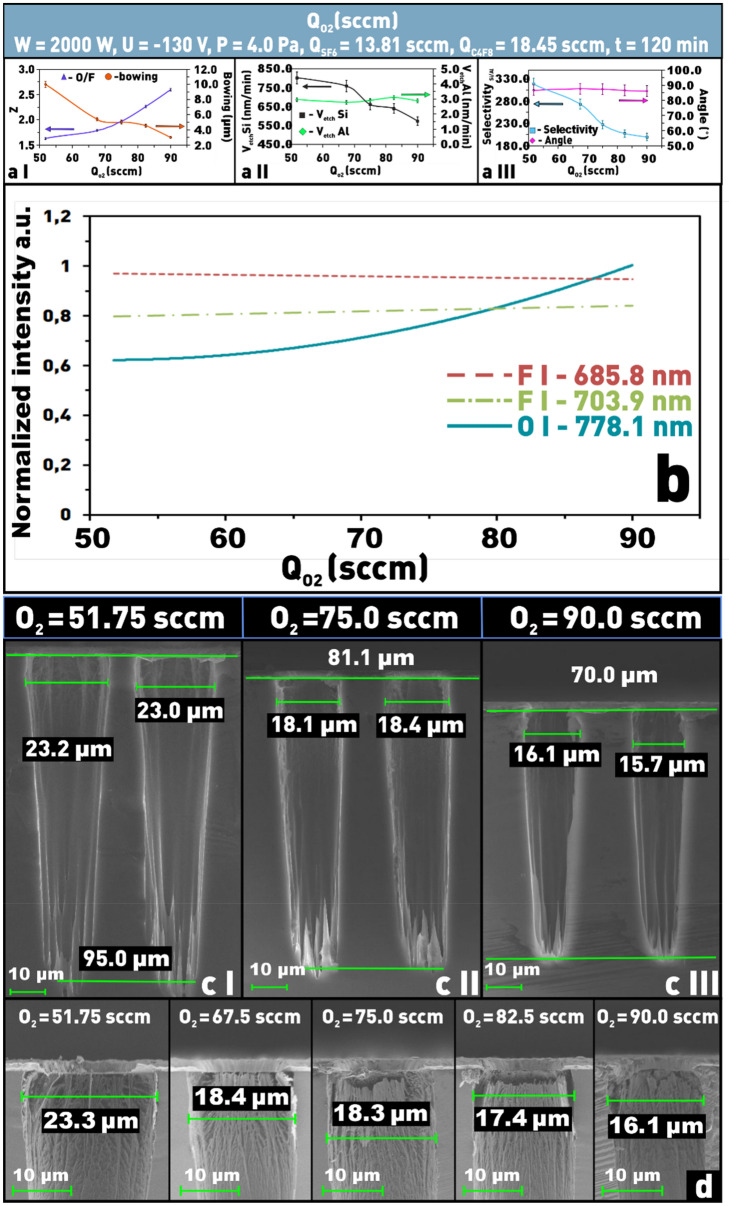
Dependences on the Q_O2_ in SF_6_/C_4_F_8_/O_2_ plasma: (**a I**) of δ and Z parameters; (**a II**) of etching rate of silicon and aluminum; (**a III**) of etching selectivity and the angle Θ; (**b**) dependences of the emission intensity of fluorine lines (685 8 and 703.9 nm) and oxygen (778.1 nm) as a function of the Q_O2_; (**c I**–**III**) microphotographs of the etching windows profiles at different oxygen contents; (**d**) microphotographs of etched windows at different contents of O_2_ in SF_6_/C_4_F_8_/O_2_ plasma to evaluate the structure overetching (δ).

From the graph of the silicon etching rate dependence on the O_2_ flow rate (Q_O2_) (Fig. 7a II) we can see that the Si etching rate monotonically decreases from ≈ 800 to ≈ 570 nm/min as Q_O2_ increases. As can be seen from Fig. 7-b, with increasing Q_O2_ there is an increase of oxygen line intensity (778.1 nm), but the intensity of fluorine lines (658.8 nm and 703.9 nm) almost does not change (Z parameter value increases), which indicates that the decrease of Si etching rate is mainly due to the increase of the protective polymer layer thickness and not to overdilution of the total gas mixture. For the same reason, δ decreases more than by a factor of 3 (from ≈ 10 to ≈ 3 μm, Fig. 7a I,c I–III,d). The silicon etching selectivity with respect to the aluminum mask decreases from ≈ 320 to ≈ 200 (Fig. 7a III). This can be due to the fact that in this series of experiments the Si etching rate decreases against of almost unchanged Al etching rate (Fig. 7a II).

Thus, a study of the influence of the gas discharge chemistry on the etching process parameters showed that the Z parameter always shows an inverse trend with respect to such parameters as the Si etching rate, selectivity, and the “bowing effect” value, independently of how the gas mixture composition varied.

### Influence of pressure in the reaction chamber

The pressure in the reaction chamber significantly affects both the intensity of ion bombardment of the substrate surface and the number of chemical reactions occurring on the surface of the treated material. As the gas pressure increases, the mean free path of the particles decreases and, therefore, the energy of the ions bombarding the surface will also decrease. At the same time, the width of the angular distribution of ions will increase^19,21,39–41^. In addition, at fixed values of gas mixture flow rate, increasing the pressure leads to increasing the dwell time of CAP at the processing surface, hence, the number of chemical reactions of CAP with Si will increase. Thus, the contribution of the physical part (ion bombardment) to the etching process can be expected to decrease and the contribution of the chemical part (etching and passivation) to increase.

**Figure 8:**
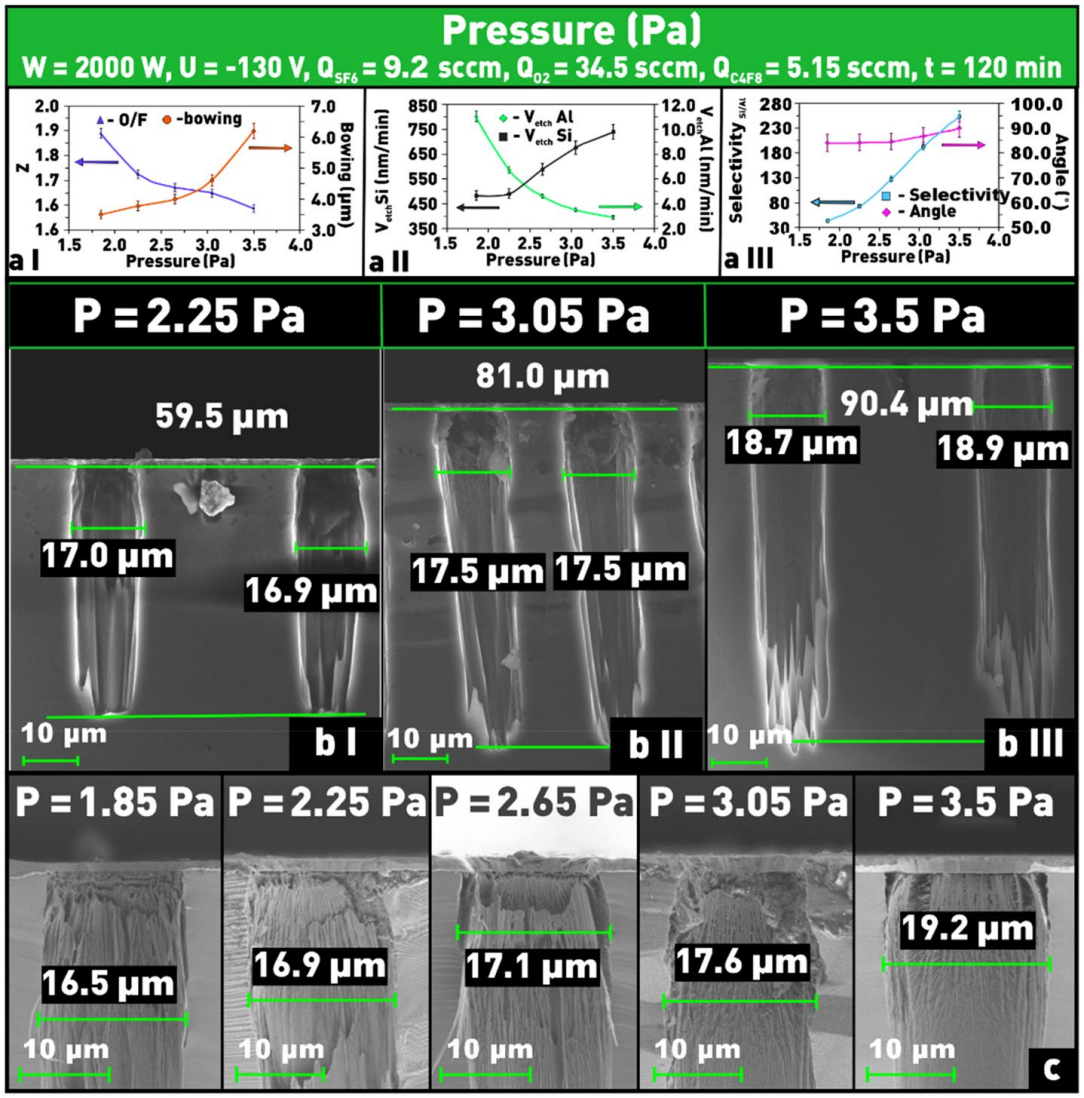
Dependences on pressure in the reaction chamber: (**a I**) of δ and Z; (**a II**) of etching rates of Si and Al; (**a III**) of etch selectivity and angle Θ; (**b I**–**III**) microphotographs of etch window profiles at different pressures in the reaction chamber; (**c**) microphotographs of etched windows at different values of pressure in SF_6_/C_4_F_8_/O_2_ plasma to evaluate the structure overetching (δ).

As can be seen from Fig. 8a II, in our experiments we observed an increase in the silicon etching rate (from ≈ 480 to ≈ 740 nm/min) with increasing pressure in the reaction chamber (Fig. 8b I–III). Since at the same time the Al mask etching rate was found to decrease (Fig. 8a II) and the parameter δ to increase (Fig. 8a I,c), the increase in Si etching rate can be related to an increase in the number of chemical reactions of fluorine radicals with Si. The decrease in the Al mask etching rate is obviously caused by an intensity decrease of ion bombardment of the surface, because as the pressure increases, the frequency of interparticle collisions in the plasma increases, the average energy of electrons and ions decreases and, consequently, the flux ratio of neutral particles to ions increases^33^. Finally, the increase in the etch selectivity (Fig. 8a III) is a direct result of the opposite trends in the etch rates of Si and Al as a function of pressure.

### Influence of bias voltage

An equally important parameter determining the etching rate is the ion bombardment. Several models of the etch process mechanisms have been proposed to explain its role^42^. First, ion bombardment assists the continuous removal of the relatively stable SiO_x_F_y_ layer formed on the silicon surface, which slowly reacts with the newly arrived fluorine atoms and holds back the etching process. Second, it is assumed that ion bombardment leads to the generation of structural defects on the silicon surface. It increases the concentration of active centers on the treated surface, resulting in an accelerated interaction of the Si surface with plasma CAP. The selectivity of silicon etching relative to the mask material is determined, to the greatest extent, by the intensity of the ion bombardment, which can lead to a rapid sputtering of the mask. Increasing the ion energy or intensity of ion bombardment increases the directionality of etching and, therefore, decreases the "bowing effect" of the structure and helps to obtain vertical structures with the inclination angle of the etched window profile wall close to 90°.

**Figure 9:**
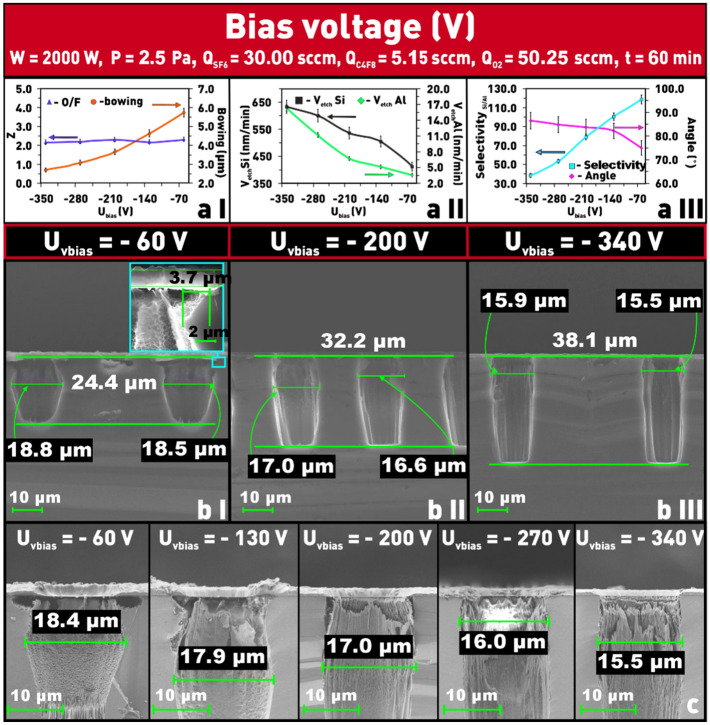
Dependences on bias voltage: (**a I**) of δ and Z parameters; (**a II**) of etching rate of silicon and aluminum; (**a III**) of etch selectivity and angle Θ; (**b I**–**III**) microphotographs of etch window profiles at different values of bias voltage; (**c**) microphotographs of etched windows at different values of bias voltage in SF_6_/C_4_F_8_/O_2_ plasma to evaluate the structure overetching (δ).

Varying the bias voltage (U_bias_) on the substrate holder makes it possible to regulate the energy of the ions bombarding the wafer surface and their movement direction toward the sample surface^28^. When a negative U_bias_ is applied to the substrate holder, the ion energy increases, resulting in increased etching rates for both the silicon and the mask (Fig. 9a II). In this case, ions with high energy strongly sputter the mask, as a result, the selectivity decreases with the increasing value of U_bias_ (Fig. 9aII–III).

Increasing the vertical relative to the lateral etch rate improves the directionality of the etch. As the U_bias_ increases (modulo), the ions fly toward the substrate with a smaller angular distribution, so the “bowing effect” of the structure, δ, decreases, and the wall angle of the etch window profile tends to 90 (Fig. 9a I, III,b I–III,c). The microphotographs (Fig. 9b I) provide clear evidence that the etch directionality has improved (the parameter δ decrease). However, even at the highest bias voltage value (− 340 V), the “bowing effect” of about 2.5 μm is observed with an etch depth of only 38 μm. This is most likely due to the faceting of the aluminum mask during Si PCE (Fig. 4)^16^. In addition, at low bias voltages (− 60 V) undercutting is observed (see the inset in Fig. 9b I), which is due to the rather large width of the angular ion distribution. A further increase in the bias voltage leads to a complete elimination of this undesirable defect (Fig. 9b II–III). It is worth noting that changing the bias voltage has no effect on the value of the Z parameter, i.e., it does not affect the ratio of oxygen and fluorine atoms in the plasma.

### Influence of applied power

**Figure 10:**
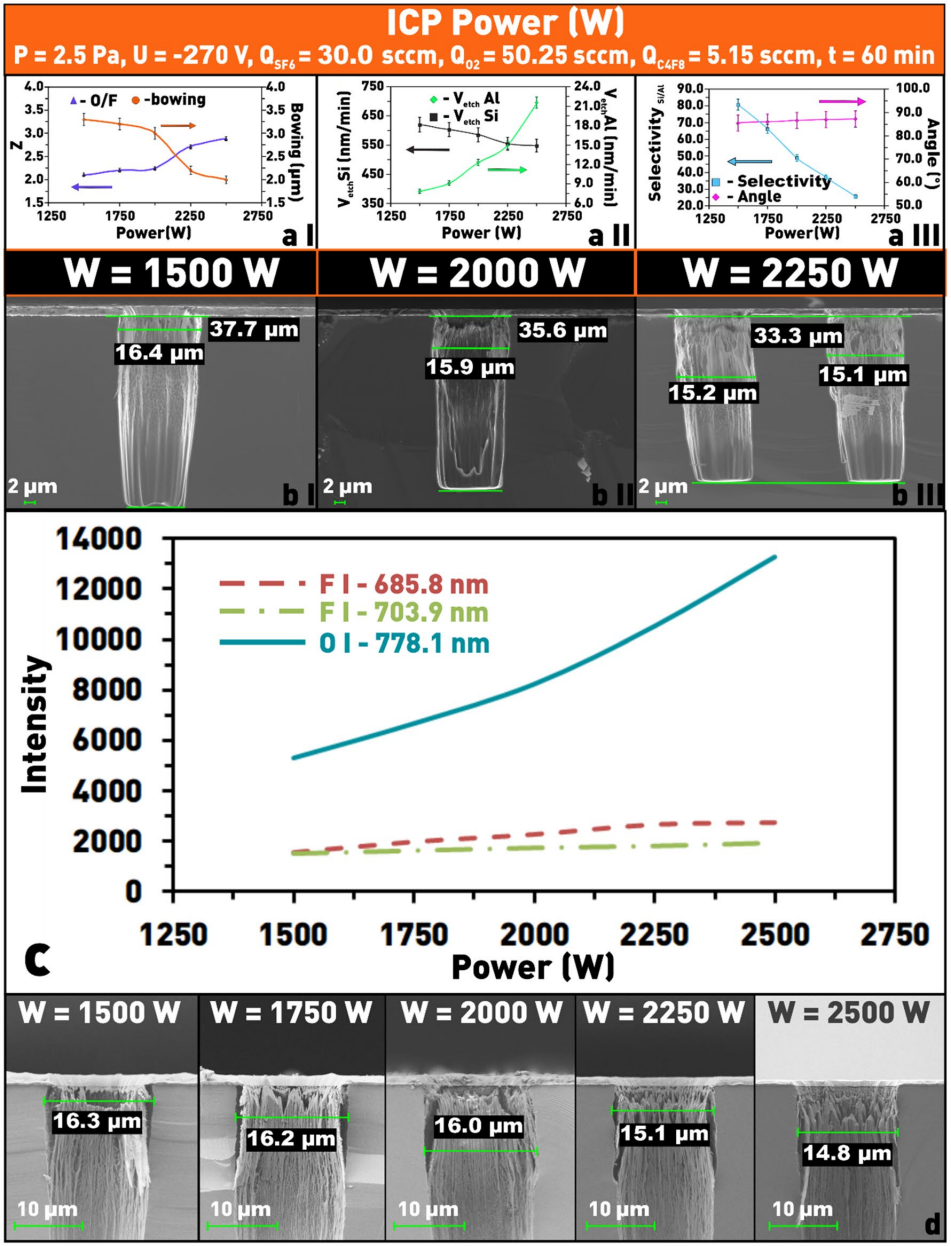
Dependences on the HF power of ICP in SF_6_/C_4_F_8_/O_2_: (**a I**) of δ and Z plasma; (**a II**) of the etching rate of silicon and aluminum; (**a III**) of the etch selectivity and the angle Θ; (**b I**–**III**) microphotographs of the etching window profiles at different HF power; (**c**) dependences of the emission intensity of fluorine lines (685.8 and 703.9 nm) and oxygen (778.1 nm) depending on the ICP HF power in SF_6_/C_4_F_8_/O_2_ plasma; (**d**) microphotographs of etched windows at different values of the ICP HF power in SF_6_/C_4_F_8_/O_2_ plasma to evaluate the structure overetching (δ).

One of the critical parameters for controlling and adjusting the PCE process is the HF power applied to the gas discharge. In the case of Si etching using gases, the dissociation of which in plasma does not form CAP that can independently, or in interaction with Si, form a protective (prevent etching) layer on its surface, increasing power, within reasonable limits, helps to increase the etching rate. However, if an etching mixture contains gases whose dissociation products can redeposit on the etching surface in the form of a protective polymer film, or form a passivating layer when interacting with the substrate material, the nature of the effect of HF power on the etching rate becomes not so straightforward. In particular, Fig. 10a II,b I–III show that in our experiments the Si etching rate decreases smoothly from ≈ 625 to ≈ 550 nm/min with increasing HF power. This etching rate trend is most likely caused by the fact that with increasing HF power the number of active oxygen particles grows faster than the number of active fluorine particles, which is confirmed by the OES data shown in Fig. 10c. As can be seen from this figure, the intensity of the oxygen line (778.1 nm) increases more than 2.5 times, while the intensities of the fluorine lines, 685.8 nm and 703.9 nm, increased only 1.8 and 1.3 times, respectively, with increasing power from 1500 to 2500 W.

As a result, oxygen radicals are more actively involved in the formation of a protective film on the sidewalls and the bottom of the structure and the passivation mechanism becomes more effective compared to etching. In addition, the selectivity of Si etching with respect to the Al mask decreases significantly (Fig. 10a III), which is associated not only with a decrease in the etching rate of Si but also with an increase in the etching rate of Al (Fig. 10a II). The reason for such a change in the etching rates of Si and Al is an increase in the number of ions bombarding not only the Si surface but also the surface of Al mask. The combination of these factors leads to a reduction of δ (Fig. 10a I,b I–III,d), i.e. to the improvement of directionality (anisotropy) of Si etching.

**Figure 11:**
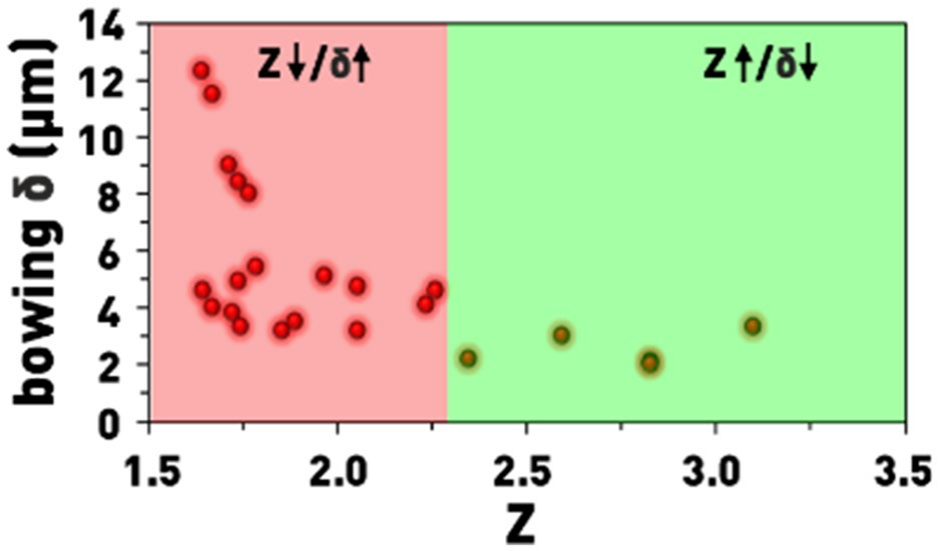
Parameter δ as a function of parameter Z.

Comparing the obtained results, we can notice the general trend for all dependencies of δ and Z on processes parameters. In all experiments these dependences show opposite trends (Figs. 5 aI, 6 aI, 7 aI, 8 aI, 10 aI), namely, increase of Z parameter value is always accompanied by a decrease of the δ parameter value, and vice versa. An exception is the case of the dependence of δ and Z on the bias voltage. In these experiments, the change in δ value occurs against a nearly unchanged value of the parameter Z. This is due to the fact that U_bias_ is the only parameter among those considered that affects only the physical part of the process and does not change the chemical composition of the plasma^43^. The observed decrease in δ with increasing U_bias_ is explained by a decrease in the width of the ions angular distribution. Although power and pressure also affect the intensity of ion bombardment, they also affect the chemical part of the Si PCE process. Thus, we can say that there is a certain relationship between the parameters Z (characterizing the ratio of oxygen and fluorine radicals in the gas discharge) and δ (quantitative characterization of the “bowing effect”). This relationship is represented as a graph in Fig. 11. The obtained dependence δ(Z) (Fig. 11) can be approximately divided into two areas. First zone with Z values ≤ 2.25, at which δ reaches maximum values up to ≈ 12.3 μm, and second zone within which Z > 2.25 and δ values do not exceed 3.5 μm.

Thus, it appears possible to control the anisotropy of single-stage Si PCE processes by focusing exclusively on the Z parameter. The side-wall angle of the etch window profile and the surface morphology can be controlled by the U_bias_, which greatly simplifies the development of deep anisotropic Si PCE technologies at various plants and facilities.

However, when selecting the optimal value of the Z parameter, the attention should be paid to the specifications (performance) of the vacuum and gas distribution systems. In the vast majority of modern systems, the pumping system includes a turbomolecular pump with pumping rates ≥ 1000 l/s (usually 1600 l/s), and the mass flow controllers which are designed for flow rates greater than 300 sccm. In this work, the vacuum system included three vacuum pumps: a turbomolecular pump (Edwards STP-301C, pumping speed 300 l/s), a Roots booster pump (ZJC70 series, pumping speed 70 l/s), and a rotary pump (2NVR-5DM, pumping speed 5.5 l/s). Thus, the limiting factor for the gas mixture supply into the chamber was the pumping rate, so that the total gas flow rate did not exceed 150 sccm. The use of more productive gas distribution and pumping systems may require some adjustment to the δ (Z) dependence definition zones boundary.

In addition, the shape (pattern) and size of the etching windows should also be taken into account, since etching windows with large and small linear dimensions have different etching behavior. It is determined by different effects that appear differently in the etching of patterns of different configurations and sizes.

Nevertheless, once the dependence δ on Z is obtained, it is possible to develop different Si etching technologies with the least effort, because by focusing on the δ (Z) dependence it is possible to get an idea of the profile geometry of desired structures even before performing a full experimental cycle aimed at the optimization the etching process.

In order to verify the proposed idea, we performed a series of experiments on etching structures with different linear dimensions of the etching window at an indiscriminate choice of process parameter values and control values of the Z parameter. The only thing we tried to minimize in this series of experiments was the angular distribution of ions and the image force (IF) effect (deflection of positively charged ions toward the sidewalls of the etch profile)^27^. Based on these considerations, the pressure in the chamber did not exceed 3 Pa, and the minimum (modulo) value of the bias voltage was − 65 V.

**Figure 12:**
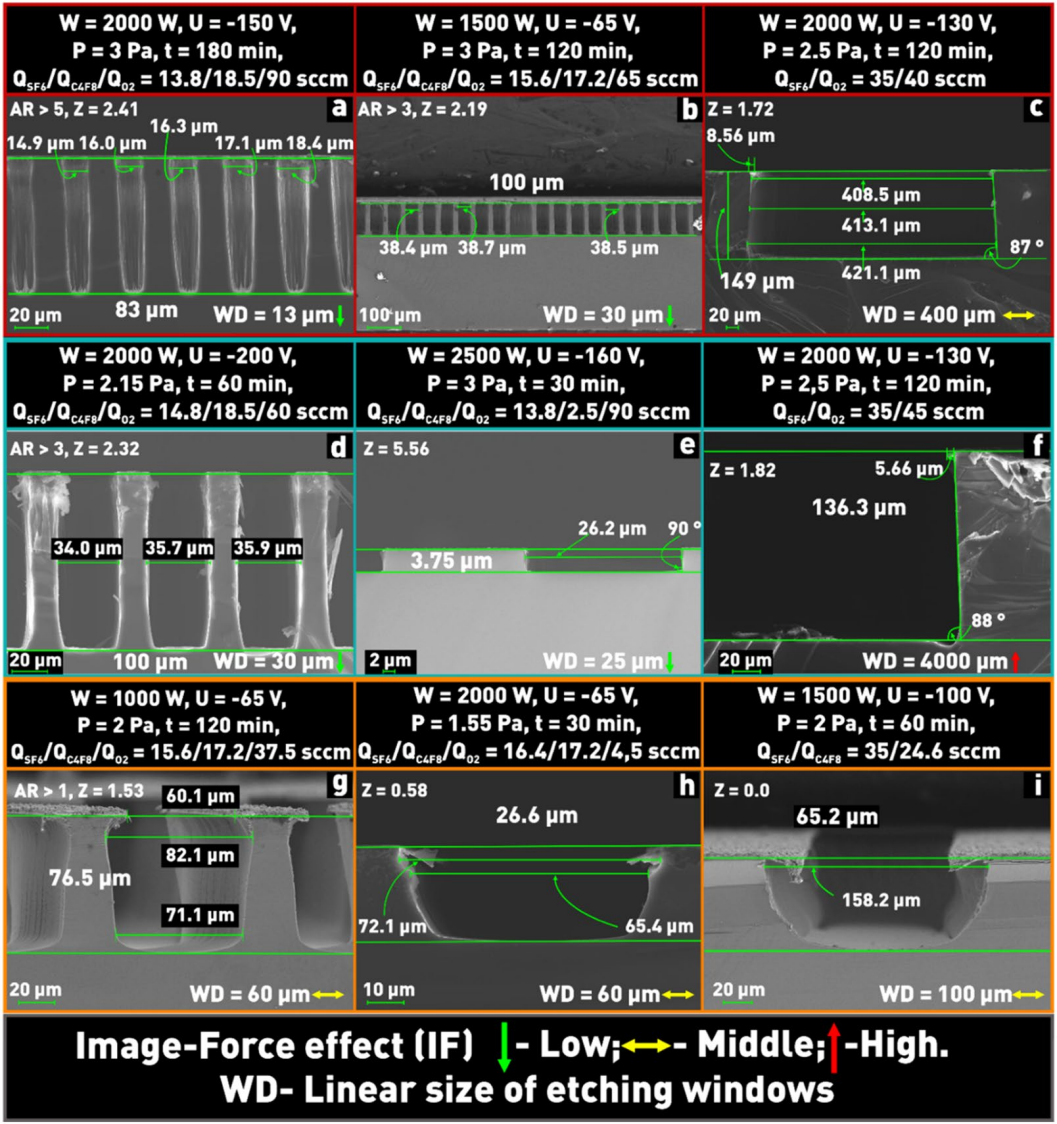
Microphotographs of etched window profiles at different values of process parameters.

The results of the experiments are shown in Fig. 12. We can see that when the Z parameter value was small enough (0 < Z < 2 for etching windows with linear dimensions up to 100 μm), i.e., corresponding to the first zone (in Fig. 11), the etching character was significantly isotropic (Fig. 12g–i). When the value of the Z parameter belonged to the second zone (in Fig. 11) the isotropy of the etch decreased significantly (Fig. 12a,b,d,e). Thus, the experiments confirmed that by controlling the Z parameter it is possible to obtain structures with etching profiles from vertical to isotropic without performing a large number of preliminary experiments aimed at determining the influence of process parameters on the etch profile.

However, it should be noted that when structures with large linear dimensions of the window are etched (Fig. 12c,f) it is necessary to make a correction for Z in a decreasing direction (in Fig. 11) to reduce the possibility of the formation of black Si at the bottom of the treated window.

Another etched structure parameter that affects the choice of the Z parameter value is the aspect ratio. The average etching rate of structures with small aspect ratios exceeds the average etching rate of elements with large aspect ratios (loading effect^27,39^). Therefore, in order to achieve a high directionality of etching structures with window sizes in the range from 13 to 100 μm it is necessary to keep the Z parameter in the range from approximately 2.2 to 2.7 and to achieve greater etching depths (over 50 μm depth) it is necessary to try to reduce the range of the angular distribution of ions to a minimum value. In the case of etching windows over 100 µm (up to 4 mm), the Z parameter should be kept in the range of 1.5 to 2.0 to minimize IF and RIE lag effects.

## Conclusions

1. As a result of this study the influence of technological parameters (SF_6_ flow rate, C_4_F_8_ flow rate, O_2_ flow rate, HF power, bias voltage, pressure) on the Si etching rate, on the selectivity of Si etching with respect to Al, on the inclination angle of the window profile wall, and on the bowing effect was determined.
2. The coefficient Z showing the ratio of the emission intensity of the oxygen line (778.1 nm) to the fluorine line (685.8 nm and 703.9 nm) was introduced.
3. The influence of the Z coefficient on the bowing effect is determined.
4. The optimum Z values for anisotropic Si structures in the size range of 13 to 100 μm (Z = 2–6) and 400 to 4000 μm (Z = 1.5–2) were determined.

These studies have shown that OES plasma diagnostics is a useful and powerful tool for monitoring the Si etching process not only to determine the endpoint, but also to control the profile of the Si resulting structures as a result of PCE in mixed mode.

However, the questions of obtaining nanoscale (less than 1 μm) structures with aspect ratios over 20 and ultra-deep structures (over 250 μm) with linear dimensions of etching windows over 1 mm remain open, using the described method to monitor the etching process. This study is planned further.

## Data Availability

The datasets generated during and/or analyzed during the current study cannot be shared at this time as the data also forms part of an ongoing study, but are available from the corresponding author on reasonable request.
